# The periosteum: a simple tissue with many faces, with special reference to the antler-lineage periostea

**DOI:** 10.1186/s13062-021-00310-w

**Published:** 2021-10-18

**Authors:** Chunyi Li, Peter Fennessy

**Affiliations:** 1 Institute of Antler Science and Product Technology, Changchun Sci-Tech University, 1345 Pudong Rd., Changchun, 130000 Jilin China; 2 AbacusBio Limited, 442 Moray Place, Dunedin, New Zealand

**Keywords:** Periosteum, Stem cells, Stem cell niche, Deer, Antler

## Abstract

Periosteum is a thin membrane covering bone surfaces and consists of two layers: outer fibrous layer and inner cambium layer. Simple appearance of periosteum has belied its own complexity as a composite structure for physical bone protection, mechano-sensor for sensing mechanical loading, reservoir of biochemical molecules for initiating cascade signaling, niche of osteogenic cells for bone formation and repair, and “umbilical cord” for nourishing bone tissue. Periosteum-derived cells (PDCs) have stem cell attributes: self-renewal (no signs of senescence until 80 population doublings) and multipotency (differentiate into fibroblasts, osteoblasts, chondrocytes, adipocytes and skeletal myocytes). In this review, we summarized the currently available knowledge about periosteum and with special references to antler-lineage periostea, and demonstrated that although periosteum is a type of simple tissue in appearance, with multiple faces in functions; antler-lineage periostea add another dimension to the properties of somatic periostea: capable of initiation of ectopic organ formation upon transplantation and full mammalian organ regeneration when interacted with the covering skin. Very recently, we have translated this finding into other mammals, i.e. successfully induced partial regeneration of the amputated rat legs. We believe further refinement along this line would greatly benefit human health.

The periosteum is a thin membrane covering all bone surfaces except for articular, tendon insertions and sesamoid bone surfaces [1]. The periosteum is firmly anchored to the underlying bone via Sharpey’s fibers [2]. The simple appearance of the periosteum belies its complexity as a composite structure that provides physical protection for the bone, a mechano-sensor for sensing mechanical loading, a reservoir of biochemical molecules for the initiation of cascade signaling, a niche of osteogenic cells for bone formation and repair, and an “umbilical cord” for the nourishment of bone tissue.

Due to differences in the nature of dynamic mechanical and biochemical environments, there is considerable variation among periosteal cells and matrix associated with the species, sex, age, embryonic origin and the site/location of the periosteum [3, 4].

In this review, we summarize the current knowledge about the periosteum with special reference to the antler lineage periostea. This review clearly demonstrates that periosteum is a type of simple tissue in appearance, but with multiple functions; antler lineage periostea add another dimension to the properties of somatic periostea, as it is capable of initiation of bony organ formation and full regeneration via interaction with the covering skin.

## The periosteum

### Histology

The periosteum comprises an outer fibrous layer and an inner cambium layer, which differ in terms of the proportions of cells, fibres and matrix. Collagen and other extracellular matrix fibers of the fibrous layer are responsible for much of the unique anisotropy and mechanical toughness of the periosteal tissue as a whole [4]. The outer fibrous layer is composed of fibroblasts, collagen, and elastin fibers. Dwek [5] further classified the outer fibrous layer into two sublayers: a superficial one and a deeper fibroblastic one. The former is generally inelastic and cell-poor, features a loose collagenous matrix with few dispersed elastic fibers, and is well-vascularized and innervated. The latter is fibroblastic, contains most of fibroblasts in the periosteum, and is rich in elastic fibers and collagens but still cell-poor with much less vascularization than the former.

The inner cambium layer is highly cellular and consists mainly of osteogenic cells at various developmental stages (quiescent, proliferating, differentiating progenitor) and osteoblasts within a much finer collagenous matrix than the fibrous layer. The more mature osteoblasts line the bone surface and less mature ones or progenitor cells are away from the bone surface on top of these osteoblasts; these less mature cells reside within rich vascular and neural networks [6]. The cambium layer provides a microenvironment that nourishes osteogenic cells allowing underlying periosteal bone formation [7].

The preferential orientation or alignment of the collagen in the periosteum is with the direction of tissue growth [8]. The collagen fibrils, perpendicular to the direction of tensile loading, degrade more easily compared to fibrils aligned with the loading direction, which is known as “strain stabilization” [9].

### Residential cell types

Periosteal cells or periosteal derived cells (PDCs) are heterogeneous in nature but consist mainly of fibroblasts, osteoblasts, mesenchymal stem cells (MSCs), mast cells and pericytes [10]. The majority of PDCs exhibit SH2, SH3, SH4, CD9, CD14, CD90, CD105 and CD166 but do not exhibit CD34, CD45 and CD106 [11–14]. The shape of the PDC may vary considerably (described as elongated, spindle-shaped, triangular or cuboidal). The concept of osteoimmunology describes the complex regulatory interactions between bone and immune cells [15]. Hence, some subtypes of macrophages are considered as integral members of the residential cell family of periosteum, two of which are highlighted here (Macrophage-lineage TRAP^+^ mononuclear cells and Macrophage-lineage F4/80^+^ cells).

#### PDCs in the cambium layer

These cells can, to a large extent, remain undifferentiated through many passages in vitro without losing their differentiation capacity [11]. Despite their common embryonic lineage, these PDCs exhibit much higher proliferation rates than bone marrow-derived MSCs (bMSCs) and maintain linear growth for more than 30 population doublings displaying long telomeres with no signs of senescence until around 80 population doublings [12, 16, 17] in a study with PDCs and bMSCs in human cells. Postnatally, PDCs exhibit greater clonogenicity, growth and differentiation capacity than bMSCs [18].

PDCs in the cambium layer of humans retain the ability to differentiate into fibroblasts, osteoblasts, chondrocytes, adipocytes, and skeletal myocytes. They are clonogenic independent of donor age and exhibit steady marker expression and growth up to 30 population doublings [19]. In contrast, bMSCs show reduced life spans in aging donors with telomere shortening and senescence [20]. PDCs from elderly people that are passaged numerous times are not only superior in producing bone or cartilage than bMSCs from a similar source, but also, surprisingly, perform much like the cells from younger people [13, 19].

PDCs in the cambium also exhibit a stronger alkaline phosphatase expression response than bMSCs to BMP2 or dexamethasone (osteo-inductive agents). PDCs and bMSCs exhibit distinct structure–function relationships and differentiation capacities attributable to their distinct milieus. For example, PDCs can be directionally induced to differentiate into osteoblasts, chondrocytes [21], myocytes [22], neuroblasts [23] and so on.

#### PDCs in the fibrous layer

Compared to PDCs of the cambium layer, the cell morphology of the fibrous layer is more typical with elongated fibroblasts [24]. Although the function of PDCs from the fibrous layer has not been reported, their main role is almost certain to be in the maintenance of the integrity of the fibrous layer through synthesis and secretion of the fibers.

#### PDCs: mast cells

Mast cells reside in the fibrous layer and synthesize and secrete the mature form of NGF (ßNGF). As mast cells are located close to the sensory nerve fibers, ßNGF secretion may play a role in the maintenance of the sensory network.

#### PDCs: pericytes

Pericytes form a distinct population in the periosteum [10]. They are polymorphic, of mesenchymal origin, and contain multiple branching cytoplasmic processes that partially surround capillaries. These cells have the ability to contract and hence can regulate blood flow in the microvasculature [25, 26]. Pericytes are in physical contact with capillary endothelial cells, with the ability to differentiate into numerous cell types, including osteoblasts [27]. With this osteoblastic differentiation potential, pericytes may serve as an ancillary source of progenitor cells [28], and may play a role in vascularization and promotion of bone formation [26]. For example, cultured pericytes of periosteal origin have been shown to mineralize in vitro and synthesize the osteoblast marker, alkaline phosphatase, as well as bone matrix proteins, including osteocalcin [27], osteonectin, osteopontin and bone sialoprotein [29], and respond to osteogenic stimuli, such as BMP and parathyroid hormone (PTH) [30]. Pericytes may have a role in fracture healing as the populations of pericytes and endogenous mesenchymal progenitors are highly correlated in an in vivo mapping study of cell fate [31].

#### PDCs: macrophage-lineage TRAP^+^ mononuclear cells

TRAP^+^ cells are abundant on the periosteal bone surface. They can induce expression of periostin and the recruitment of PDCs to the periosteal surface for bone formation and regeneration, thus playing an essential role in regulation of periosteum homeostasis, repair and regeneration. Resident TRAP^+^ cells of the periosteum are distinct from osteoclasts, and there are likely to be different macrophage subtypes that play different roles in bone formation, repair and regeneration. For example, a deficit of TRAP^+^ cells in the mouse periosteum impairs recruitment of PDCs for cortical bone formation [32]. Macrophage/monocytes differentiate into periosteal TRAP^+^ cells during bone growth and secrete PDGF-BB, which transcriptionally induces expression of periostin to create an osteogenic microenvironment in the fibrous layer of periosteum.

#### PDCs: macrophage-lineage F4/80^+^ cells (OsteoMacs)

Another discrete population of resident macrophages is also found to be distributed along the bone lining surface within the periosteum in murine and human bone, and these cells are termed OsteoMacs [33]. OsteoMacs are different from osteoclasts, although the two cell populations are related by their shared precursors and CSF-1 dependence, the F4/80 Ag is completely absent from osteoclasts [34]. These resident macrophages undergo tissue-specific adaptation and contribute to ongoing physiological processes and tissue repair [35]. Juvenile rats are found to have more Stro1^+^, F4/80^+^ cells and blood vessels and fewer TRAP^+^ cells in the periosteum than other age groups [36].

Chang et al. [33] reported that in an in vitro culture, the co-isolated OsteoMacs but not osteoblasts responded to pathophysiological concentrations of LPS by secreting TNF. OsteoMacs are required for efficient osteoblast mineralization in response to the physiological remodel stimulus, elevated extracellular calcium. Depletion of OsteoMacs in vivo in mice causes complete loss of osteoblast bone-forming surface at this modeling site. OsteoMacs are also the most obvious candidates to detect and respond to bone damage, a critical event in initiation of bone remodeling and osteoclast recruitment [37]. Overall, OsteoMacs are an integral component of periosteum and play a novel role in bone homeostasis through the regulation of osteoblast function.

#### PDCs: nestin positive (Nestin^+^) cells and leptin receptor positive (LepR^+^) cells

Both Nestin^+^ cells and LepR^+^ cells reside primarily in the outer fibrous layer of periosteum and may be subsets of PDCs responsible for periosteal bone formation, as such subsets have the potential to differentiate to osteoblasts for periosteal bone formation. In mice, Nestin^+^ PDCs are found primarily during bone development, whereas LepR^+^ PDCs are essential for bone homeostasis in adults. Both Nestin^+^ PDGFR-α^+^ and LepR^+^ PDCs of the periosteum form more CFU-Fs than do bMSCs [32].

### Blood supply

The periosteal circulation is an important component of bone vascularization. The blood supply of the periosteum is derived from four vascular systems, namely the intrinsic periosteal system, periosteocortical anastomoses, and the musculoperiosteal and the nutritive periosteal systems [7]. The periosteum has a rich vascular plexus and is regarded as the “umbilical cord of bone” [38], and provides at least one-third of the blood supply to the cortical bone, with the remainder from the intramedullary niche [29, 39]. The blood vessels lie mainly within the fibrous layer of the periosteum.

Blood vessels in human tibia periosteum exhibited a ring pattern [40] whereas those in the dog tibia periosteum showed a longitudinal pattern [41] although both systems can co-exist in the same bone [7]. In addition, there are some bones with a system of short vessels which are connected by small vessels to the circular and longitudinal systems. These short vessels supplied by the musculo-periosteal vessels are found where there is a fleshy muscle attachment [7].

### Innervation

The nervous system has emerged as an important regulator of bone metabolism through central control of the osteogenic cell activities of the periosteum [42]. Osteoblasts play a role in switching the phenotype of sympathetic fibers from an adrenergic to a cholinergic state during establishment of innervation of the sternum periosteum [43].

The adrenergic sympathetic nervous system controls bone formation and resorption mostly through the ß2-adrenergic receptors (AdB2R) in the appendicular and axial skeletons [44]. AdB2R activation up-regulates RANKL (an activator of resorption) by osteoblasts [45]. Enhanced NGF expression increases innervation while NGF depletion results in sympathetic hypo-innervation [46]. The cholinergic nervous system controls storage of immature nerve growth factor (proNGF) in the extracellular matrix and Sema3a expression by osteogenic cells and osteocytes. Mature NGF (ßNGF) is expressed only in mast cells residing away from the bone surface close to the vessels irrigating the site; this population is involved in the control of osteoclast precursor entry in the periosteum [47].

Destruction of the sympathetic system induces mast cell activation and ßNGF release to the extracellular milieu, suggesting that factors synthesized by the sympathetic fibers stabilize mast cells. In the non-osteogenic compartment of the periosteum, treatment with vasoactive intestinal peptide (VIP) decreases the ratio of activated/total ßNGF^+^ mast cells [42]. VIP-immunoreactive fibers (IR) are located along the interface between the cambium and fibrous layers of the periosteum, and VIP is a pleiotropic peptide with neuroprotective actions [48, 49]. Sensory nerve fibers also release calcitonin-gene related peptide (CGRP) that is trophic for osteoblasts [50].

### Factors that influence attributes of the periosteum

Periosteum throughout the bone surface is not uniform in structure, cell population and the function, but appears to vary considerably in different age, location, sex, embryonic origin and species.

#### Age

The cambium layer of the periosteum is at its thickest in the fetus and becomes progressively thinner with age. In the adult, it is so thin that it cannot be readily distinguished from the outer fibrous layer [51]. Blood vessel density and the number of periosteal fibroblasts also decrease with age so that in the adult, the periosteum is evident only as a very thin tissue layer enveloping the bony structures [28]. Although old age does not seem to inhibit the regenerative properties of the periosteum, some age-related changes include a decrease in periosteal fibroblast number, fibrous layer thickness, osteoblast number, collagen formation, osteoid zones and vessel density [10, 17, 52]. In this respect, age-related degeneration (decrease in thickness and cell number) is observed in the diaphyseal periosteum in aged rats [53]. Both TRAP^+^ mononuclear, F4/80^+^ and Nestin^+^ cells, all abundant on the periosteal bone surface in young mice, decreased markedly during late adulthood, whereas LepR^+^ cells were abundant in adult mice [32].

A higher percentage of Stro-1^+^ cells are found in the diaphyseal and metaphyseal periosteum in juvenile rats; in mature and aged rats, however, Stro-1^+^ cells are significantly less and the intensity of Stro-1 staining is weaker compared with the juvenile group, indicating the highly osteogenic/chondrogenic nature of periosteum in young animals [36]. Numerous macrophages, but a limited number of osteoclasts, are found in the periosteum especially in the cambium layer of juvenile rats. In aged rats, however, both macrophages and osteoclasts are increased.

Both the cambium and fibrous layers in the periosteum of juvenile rats are well vascularized, while in mature rats, blood vessels are predominantly in the fibrous layer. The higher degree of vascularization in the periosteum of juvenile rats suggests a role in nutrient and osteoprogenitor cell supply. Both the thickness and number of cells in the diaphyseal periosteum decreased with age [36]. Interestingly, PDCs from the aging human retain high growth potency and differentiation capability, although their capacity to differentiate toward chondrogenic and adipogenic lineages diminishes with age [54].

Overall, periosteum in bone formation, repair and responsiveness to hormones and cytokines declines with age, although the potency of osteogenic differentiation of the PDCs may be maintained.

#### Location

Besides age, changes in periosteum are also site-dependent, with differences in periosteal anatomy or activity evident throughout the skeleton. The morphology of the periosteum is highly variable between bones within an individual and even within bones [55]

The specific site plays a key role in the properties of the periosteum. For example, the rate of periosteal bone formation differs more than three-fold through the skeleton in rats [36]. The osteogenic potential of bovine periosteum (from young calves) was highest in the cranium and decreased through the ilium, radius and the mandible; notably, the cranium and mandible are characterized by intramembranous ossification while the radius and ilium exhibit endochondral ossification. In other work using periosteal free grafts, the calvarial periosteum had less osteogenic potential than that of the tibia [56, 57].

In in vitro studies of chondrogenic potential of the periosteum, the ilium, scapula, and tibia gave rise to chondrogenic PDCs whereas PDCs from the skull exhibited no signs of chondrogenesis [58, 59]. Osteogenic activity of periosteal cells is more pronounced in flat than in long bones [60], while others [55] reported that mechanical characteristics of periosteum are different between metaphyseal and diaphyseal regions and between periosteum harvested from the anterior, medial, lateral, and posterior aspects of tibia.

In summary, there is evidence of considerable variability in the osteogenic and chondrogenic capacity of the periosteum depending on the location but regardless of the ossification pattern (intramembranous or endochondral). The appropriate choice of periosteum for bone and cartilage tissue engineering and regeneration should be a function of the specific bone to be utilized [3].

#### Embryonic origin: mesoderm vs neural crest

There are two populations of adult PDCs that can be distinguished based on their embryonic origins: mesoderm-derivative and neural crest-derivative [61]. The facial skeleton is derived exclusively from neural crest, whereas the rest of the skeleton is derived from mesoderm [62, 63]. From a histological perspective, the healing of a cranial neural crest-derived skeletal element is no different from healing in a mesoderm-derived element. Both can contribute to both endochondral and intramembranous bones [64]. The PDCs, however, have ‘positional memory’, which influences how the cells behave when grafted into ectopic locations. When the neural crest-derived bone is injured, the callus is composed entirely of neural crest-derived cells, whereas when the tibia is damaged, the injury site is occupied entirely by mesoderm-derived cells [61]. The findings of two distinct populations of PDCs would have clinical implications: should bones preferentially heal using cells of the same embryonic origin, then repair strategies should take this into consideration to ensure maximal benefits. Indeed, most grafting procedures for craniofacial defects use mesoderm-derived cells (e.g. the fibula, iliac crest, ribs), which has been found to be less effective than grafts of neural crest-derivatives [65].

#### Sex

Sexual dimorphism within species is common, and hence the sex of the donor from whom cells are obtained may be expected to affect the biology of PDCs; for example, PTH and estrogen have been shown to affect proliferation and apoptosis of PDCs from different sex-origin [59].

Animal studies support a positive effect of androgens and a negative effect of estrogens on the rate of periosteal bone formation [66]. At puberty in males, the periosteum expands due to androgens with little change in the endocortical (medullary) diameter such that cortical width increases; in females, periosteal expansion ceases, and the medullary diameter decreases as endocortical bone formation occurs. A role for insulin-like growth factor 1 (IGF-1) in the regulation of periosteal apposition during puberty has long been postulated, especially in concert with sex steroids [67].

#### Species

PDCs in different species are also different. For instance, rabbit-derived PDCs (rPDCs) are smaller than human PDCs (hPDCs) under the same culture conditions [68] with striking differences reported in the osteogenic capacity of rPDCs and hPDCs. In vitro, rPDCs grow faster and reached higher cell density than hPDCs at the confluent stage. In vivo, hPDCs give rise to extensive bone formation, whereas rPDCs fail to form bone. In the initial stages, PDCs of both species show high osteogenic potential. However, in the later stages, the cell response favors resorption of new bone tissue from rPDCs but do not affect bone tissue formed from hPDCs [69].

### Function

As known from orthopedic practice, destruction of the periosteum leads to delayed bone healing or nonunion [70]. The periosteum has the ability to heal large, critical sized (unable to bridge on their own) defects in both long and flat bones [71]. Periosteal tissue has been used very effectively in the enhancement of bone formation in dentistry and maxillofacial reconstruction [72, 73]. Periosteal cells contribute to bone repair by recapitulating specific features of the bone development process [74, 75]. In this respect, the cambium layer of the periosteum is capable of: (a) forming normal lamellar bone apposition on cortical bone that grows in width, and (b) forming primary, woven bone after a fracture. The periosteum has been shown to act as a niche for many types of cells that participate in both endochondral and intramembranous ossification during prenatal development and postnatal fracture healing [76, 77].

#### Bone formation

During natural bone growth in young people, the cambium layer of the periosteum expands with an increasing girth and length of bones [78]. In long bones, there is longitudinal growth through endochondral ossification of the diaphysis and parts of the metaphysis; there is radial growth through direct apposition of cortical bone by PDCs from the inner cambium layer of the periosteum (intramembranous bone formation). Apposition of bone around and between periosteal vessels results in formation of periosteal ridges, which, in subsequent phases unite around periosteal vessels thus producing Haversian canal osteons [29] surrounded by concentric rings (lamellae) of matrix, in so doing lamellar bone is formed.

Although remodeling of trabecular bone occurs in the bone marrow microenvironment, and growth and modeling of cortical bone takes place in the periosteum, the periosteum provides a supportive microenvironment with vasculature, nerves, PDCs, and osteoprogenitors, which resembles a unique bone marrow for the growth and modeling of cortical bone.

#### Bone repair

Skeletal repair is a dynamic and well-orchestrated process that involves complex and spatiotemporally coordinated function of different cellular compartments and integrated molecular pathways. Immediately following cortical bone injury, the periosteum undergoes a series of changes that initiate endochondral and intramembranous bone formation at the site of injury.

PDCs near the cortical bone injury site differentiate into chondroprogenitors whereas PDCs at the periphery of the cortex furthest away from the site adopt an osteogenic cell fate. The periosteum is the major contributor to cartilage and bone repair within the callus, whereas cells within the local bone marrow and endosteum form bone within the bone marrow cavity and do not migrate out of the marrow to form the callus [74]. The periosteum stabilizes bones mechanically during fracture healing. The inflammatory phase is believed to stimulate mesenchymal cell migration and proliferation. Following inflammation, mesenchymal cells aggregate at the repair site and differentiate into chondrocytes and osteoblasts. Collagen matrix is secreted and subsequently mineralized. These events result in the formation of a soft callus that bridges the two fracture ends. With time, the soft callus continues to ossify, and woven bone is formed. Eventually, upon remodeling, the original shape and structure of the bone will be restored [79, 80].

Periosteum-derived Prx1^+^ (paired-related homeobox gene-1^+^) PDCs contribute to cartilage and bone within the callus, while bMSCs have less potential to form cartilage and do not participate in forming new bone at later stages. The majority of bMSCs stay at the periphery of the callus and PDCs integrate far into the callus and cartilage by day 10 [18]. In contrast to bMSCs, which are restricted to the bone marrow compartment during bone repair and indirectly stimulate healing via the secretion of growth factors [74], PDCs are directly involved in bone repair [18]. Thus, PDCs have been used to generate in situ bone tissue for fracture healing or bridging of critical-sized defects in combination with various scaffolds [81, 82]

### Regulation

#### Biochemical factors

##### Wnt, BMP and Hedgehog

Histologically distinct layers of the periosteum are associated with distinct molecular expression domains. Multiple factors and transduction pathways are involved in the regulation of periosteum functions.

Canonical Wnt signaling is heavily involved in regulation of the periosteum. Wnt signaling is an upstream regulator of BMP signaling in osteoblasts [83] and multiple Wnt proteins, as well as their modulators, are expressed in periosteum [84]. Delivery of a Wnt/β-catenin inhibitor, DKK1, can suppress bone repair whereas administration of a DKK1-neutralizing antibody improves the effects of repair and regeneration [85]. In this respect, early periosteum-lineage cells lacking β-catenin are blocked in osteoblast differentiation but develop into a chondrocyte phenotype instead [86, 87] and increased β-catenin activity is evident in osteoblasts lining the periosteum throughout fracture healing in mice. This signaling has distinct roles in PDCs and committed osteogenic progenitor cells, namely: (1) inhibiting PDCs from differentiating into adipocytes [88]; and (2) committing PDCs to the osteoblast lineage [89].

Several factors related to the BMP pathway have been identified in the activated periosteum including BMP-2, -3, -4, -5, -8, noggin, BMPRIA, BMPRII, and pSmad 1/5/8 [90]. Deletion of BMP-2 in periosteum abolishes fracture callus formation, suggesting a critical role of BMP-2 in the initiation of repair [91]. BMP-2 appears to be at the apex of the BMP signaling cascade that initiates cellular proliferation and differentiation of PDCs during repair and regeneration. BMP-2-induced osteogenic differentiation of PDCs of the periosteum might be initiated via upregulation of the osteogenic transcriptional regulators Runx2 and Osx, which are consistent with BMP2 expression peaking at around fourfold that of basal expression one day after fracture [92, 93]. In the early stages of fracture repair, expression of BMP-2/-4 and BMP-7 were strongly induced in the thickened periosteum near the fracture ends, coinciding with an enhanced expression of the BMP type II receptor [94]. COX-2 is one of the important downstream mediators of BMPs and coordinates with BMPs in differentiation of PDCs [95]. Deletion of COX-2 globally or specifically significantly impairs proliferation of PDCs and delays subsequent repair either through endochondral or intramembranous ossification [96, 97].

TGF-β is synthesized at high levels in the periosteum during fracture healing, enhances the proliferation and differentiation of PDCs, increases production of extracellular matrix and is chemotactic to bone cells [98]. TGF-β cooperates with Wnt signaling in osteoblast differentiation, activates β-catenin signaling via the ALK5, Smad3, PKA, and PI3K pathways, and modulates osteoblastogenesis [99].

In the early stages of fracture healing, Hedgehog signaling is activated for efficient periosteum-mediated repair and regeneration and is enhanced in early periosteal callus formation. Activation of Hedgehog signaling promotes osteogenic and chondrogenic differentiation of PDCs in synergy with BMP-2. Postnatal deletion of Smo, a receptor that transduces all Hedgehog signaling, impairs osteogenic differentiation of PDCs in vitro, and results in a halving of periosteal bone callus formation in vivo [100]. Hedgehog, most likely Indian Hedgehog (IHH) signaling, plays a key role in proliferation and differentiation of PDCs at the early stage of endochondral bone repair. Compared to bMSCs, PDCs are more responsive to BMP-2 and Hedgehog agonists, suggesting its unique role in bone repair and regeneration.

IHH is expressed in the nascent cartilaginous tissues in the periosteal callus adjacent to the bone surface at the initiation stage of healing. The periosteal markers, β-tubulin, Type V collagen, RAI14, Decorin and YBX-1, are all expressed in the cambium layer of the periosteum [101], and YBX-1 is a translational repressor protein [102]. Deletion of Smoothened (receptor of IHH) results in a halving of the size of the bone callus and significant reduction of PCNA^+^ cells around hypertrophic chondrocytes in Smo-deleted periosteal callus, suggesting a role of IHH in driving the expansion of callus formation during the early stage of endochondral bone repair [100].

PTHrP is a small polypeptide [103], and acts as a paracrine regulatory molecule predominantly, while PTH acts as a classical systemic peptide hormone [103, 104]. PTHrP expression is evident in the fibrous layer of the periosteum [105]. PTH/PTHrP receptor is strongly expressed in the periosteum at the site of the fracture by day 3 following fracture [106]. PTHrP and IHH seem to play complementary roles in fracture healing, and there is functional cross talk integrating BMP and PTHrP/IHH signaling in regulation of osteoblastic differentiation and proliferation during the bone healing process. PTH therapy (and even mild hyperparathyroidism) may increase bone size and strength through complex effects on bone forming elements on the periosteal surface [107].

##### NGF and Sema3a

NGF is a trophic factor for nerve fibers and is also involved in differentiation and survival of the osteogenic cells [108]. The two forms of NGF, proNGF and ßNGF, have various roles in periosteum metabolism. Their expressions are well segregated: proNGF is released and stored in the extracellular matrix of the cambium layer, while ßNGF is expressed only in the fibrous layer [42]. Cells in the cambium layer metabolize the proNGF for their survival and continue to synthesize the factor, probably at a lower rate since NGF is secreted in concentration proportional to nerve fiber density [109]. A strict compartmentalization of the different forms of NGF appears to regulate periosteum sensory and sympathetic nervous system homeostasis, with proNGF being associated with the sympathetic system and ßNGF controlling the sensory system.

Semaphorin 3a (Sema3a) is a molecule that promotes osteoblast differentiation, beside its repulsive role on sympathetic and sensory fiber networks [110]. However, Sema3a derived from sensory nerve fibers influences osteoblast metabolism, although not that derived from osteogenic cells [111]. Sema3a has bifunctional effects on bone metabolism: besides its action on osteogenic cells, it inhibits osteoclast differentiation and is repellent for osteoclast precursors [110]. While VIP regulates Sema3a expression by osteogenic cells, Sema3a may restrain and counterbalance the pro-resorption action of VIP [112], in synergy with CGRP whose expression by PDCs varies with VIP release or inactivation [42]. Interactions between sympathetic nerve fibers and osteogenic cells in the mandibular periosteum locate the VIP-IR fibers at the periphery of the cambium layer of periosteum and prevent its penetration by sensory never fibers. At this site, VIP-elicited expressions of NGF and Sema3a participate in the trophic maintenance of the osteogenic cells and in prevention of hazardous resorption by possibly regulating the number of preosteoclasts and by protecting the bone surface by repelling them.

##### IGF family and HIF-1α

The complexes of ligand and the receptor of insulin-like growth factor (IGF) family increase locally in the fracture callus of human patients, and their expression is markedly increased in the PDCs of multi-layered periosteum in the developing bony calluses [113, 114]. The binding of IGF-1 to its receptor (IGF1R) triggers the activation of several intracellular kinases, including phosphatidylinositol-3-kinase (PI3K). The latter activates protein kinase B (AKT) [115, 116]. Active AKT accelerates PDCs/osteoblast differentiation through two transcription factors, Runx2 and osterix, which acts via synergy with the Wnt-β catenin pathway [117].

HIF-1α plays dual roles in signaling during bone regeneration via periosteum: (1) HIF-1α is necessary for increased VEGF production to mediate the angiogenic response during bone repair; (2) HIF-1α-dependent adaptations in glycolysis and mitochondrial metabolism ensure cell survival during the early stages preceding the arrival of the blood vessels [118].

##### Periostin

Periostin is the only protein that is present in a higher amount in periosteum than in other bone locations [119]. Periostin is involved in the regulation of periosteum homeostasis [18], and deletion of the gene impairs PDC functions and fracture consolidation. Periostin-deficient periosteum cannot reconstitute a pool of PDCs after injury and hence contribute to healing after bone injuries resulting in severe repair defects. Similarly, the periostin KO phenotype is not due to deficient proliferation, but to the inability to maintain a pool of PDCs in the periosteum. Periosteum contains PDCs that can self-renew during several injury cycles and periostin is required for this self-renewal capacity by regulating the periosteal niche of PDCs. Thus, periostin is a key regulator of PDCs in periosteum and their niche.

The synthesis of periostin is increased four-fold during the first 3 days after the fracture; by day 14 periostin is expressed at the junction between hypertrophic cartilage and bone, and by osteoblasts and osteocytes in the new bone matrix; by day 28 periostin is detected in newly formed periosteum at the periphery of the ossified callus [120]. Deletion of periostin in the PDCs impairs their osteogenesis and adipogenesis compared to the wild-type PDCs in vitro, although their chondrogenic potential is not affected. In response to bone injury, periostin and other ECM proteins linked to periostin are upregulated in PDCs and periostin is crucial for adequate bone repair [18].

Conditional ablation of platelet derived growth factor (PDGF)-BB in macrophage-lineage TRAP^+^ cells reduced periostin expression in the periosteum [32]. Mechanistically, PDGF-BB upregulates periostin expression via induction of the phosphorylation of PDGFR-β, PI3K, AKT, and CREB. CREB is essential for enhanced osteogenesis, which is modulated by PI3K/AKT signaling [121]. PDGF-BB induces direct binding of pCREB to the periostin promoter. Periostin expression, induced by TRAP^+^ mononuclear cells, maintains the periosteal microenvironment and regulates differentiation of PDCs for periosteum homeostasis and osteogenesis [32].

#### Mechanical factors

In addition to biochemical induction of lineage commitment, studies have shown PDCs exhibit exquisite mechano-sensitivity, including lineage commitment independent of biochemical factors. In this respect, mechano-induction is much easier to control both spatially and temporally than biochemical signals. Periosteum is a highly specialized, mechanosensitive tissue. The native environment of PDCs is mechanically regulated by a combination of tension and shear. Mechanical force applied in vivo induces the expression of a variety of genes in the periosteum [122] and a rapid transformation of quiescent periosteal surfaces to those on which bone formation occurs [123]. In fact, it has been suggested that the mechanical loading environment is a primary modulator of periosteal apposition growth on bones [124].

The periosteum may exhibit directionally-dependent permeability and permeability is highly dependent on the stress-state of the tissue [125]. Gross mechanical manipulation of the periosteum, such as extra-periosteal solution injections and surgical release of the periosteum, stimulates periosteal hypertrophy, DNA synthesis, cell proliferation, and bone growth [126, 127].

## The antler-lineage periostea

### Background

**Fig. 1:**
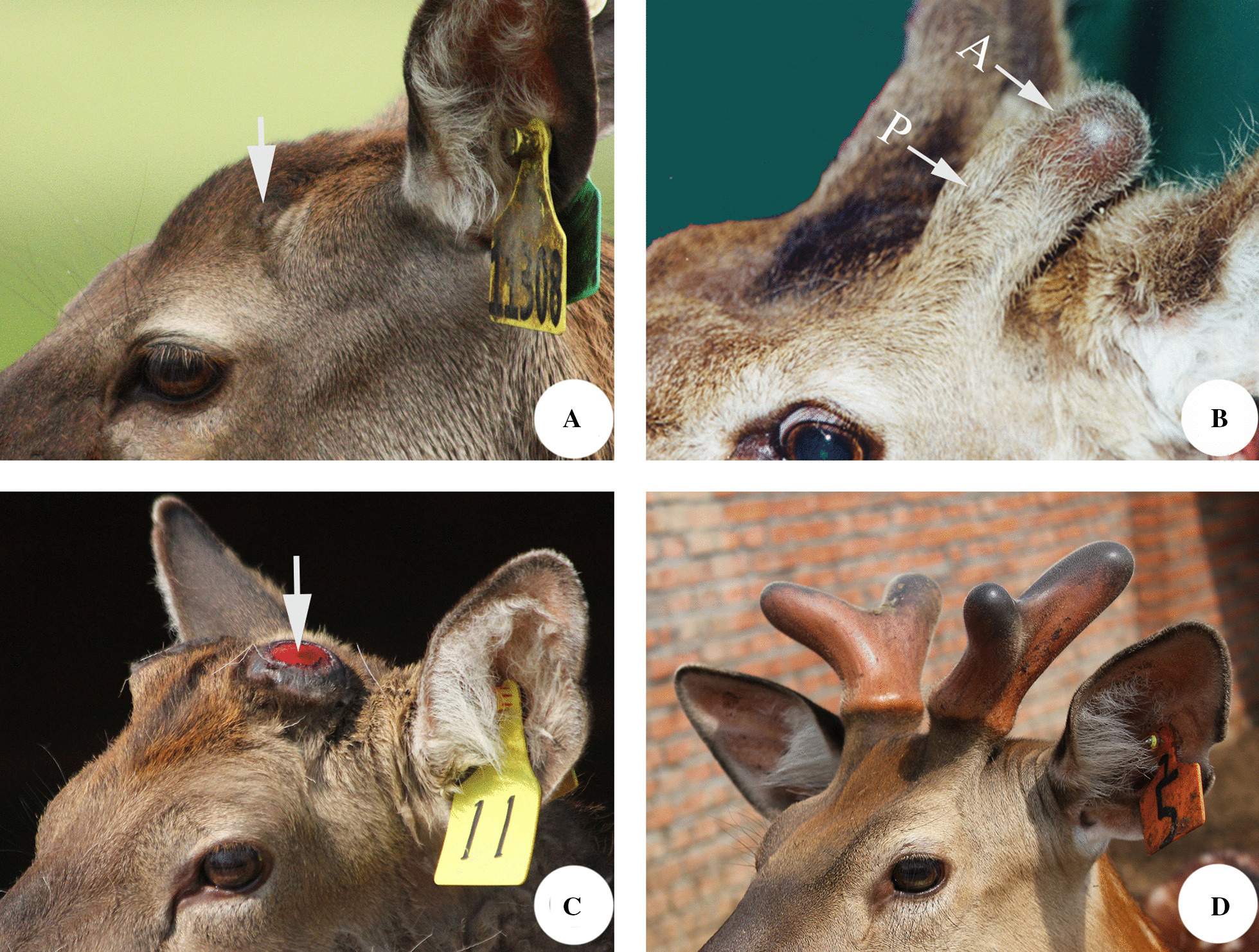
Morphology of generation of pedicles and first antlers, and regeneration of antlers. **a** Frontal crest (arrow), the presumptive pedicle growth region, in a prepubertal male deer; **b** Full grown pedicle (P) with a newly emerging antler bud (A), note that the pedicle is enveloped with typical scalp skin and the antler with a special type of skin, known as velvet skin; **c** A fresh wound (arrow) on top of a pedicle created following casting of the previous hard antler; **d** A two pair of 2-branch-antlers regenerated following the wound healing over the pedicles

Deer antlers are the only mammalian organs that can fully regenerate once lost [128]. Before an antler can grow, a permanent bony protuberance, known as pedicle (the antler antecedents), must firstly form from the frontal crest (Fig. 1A) of a male deer head; antlers can then develop from the apices of fully-grown pedicles (around 5–6 cm in height in red deer; Fig. 1B; [129]. Deer are not born with pedicles (although presumptive pedicle tissue is evident in utero at around day 100 pregnancy, [130, 131]). However, the pedicles start to grow when the animal approaches puberty due to the elevation of circulating androgen hormones [132, 133]. The first-formed antlers grow rapidly, calcify fully, shed their velvet skin and are then cast in the following spring to initiate subsequent full antler regeneration. From then on, development of the regenerating antler enters a well-defined cycle: the hard antler is cast (Fig. 1C), the wound heals, the velvet antler regenerates and grows (at a phenomenal rate: up to 2 cm/day) through spring (Fig. 1D); calcification follows (at intensive speed: up to 250 g/day) and the velvet skin is shed in autumn; the firm attachment to the living pedicles is maintained through winter, and the hard antlers are then cast from the pedicles in the next spring triggering a new round of antler regeneration [128]. There is some variation with the relative timing of casting and regrowth among species but in the Cervidae (*Cervus elaphus*), the casting is followed immediately by healing and regrowth.

**Fig. 2:**
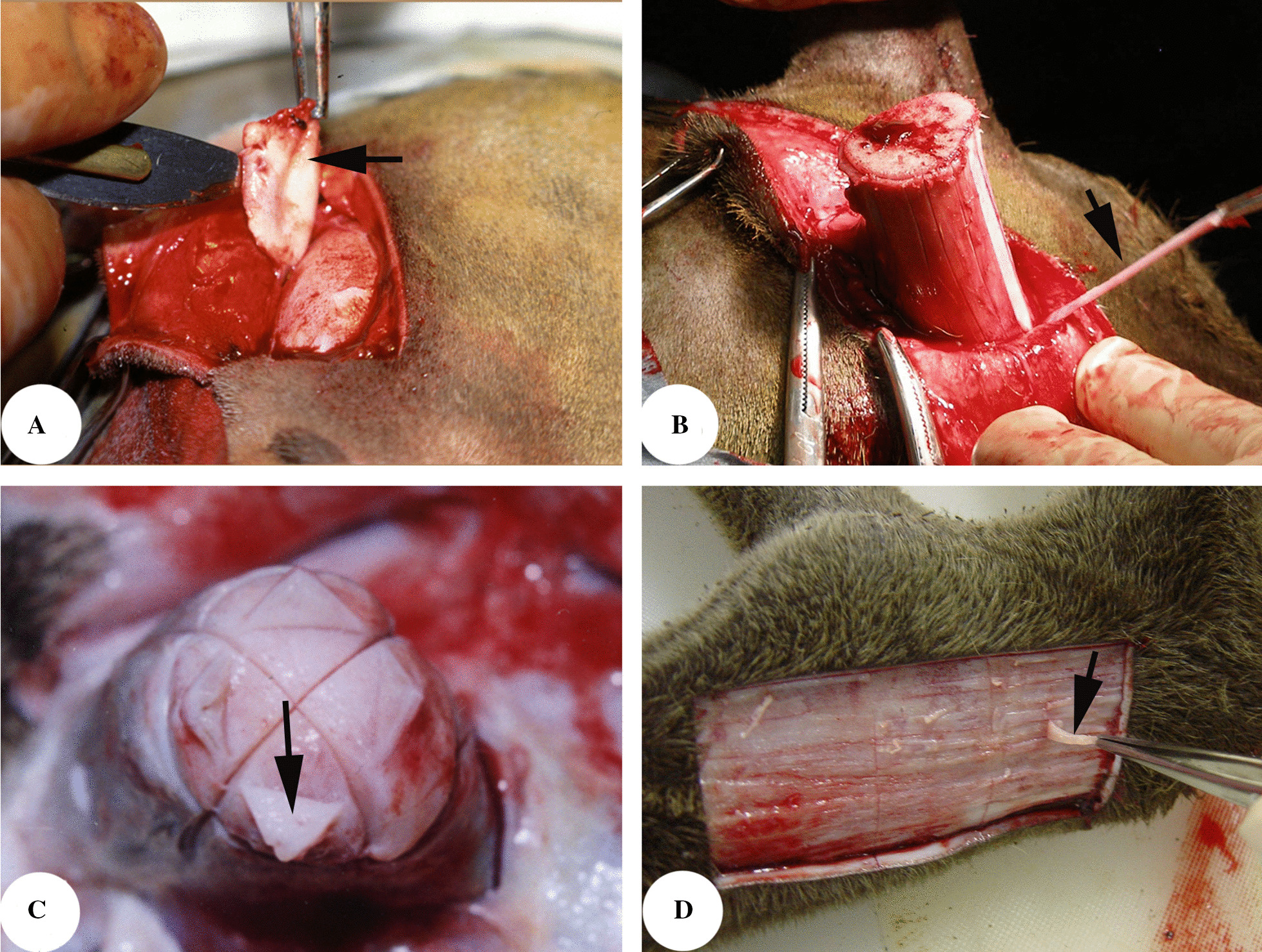
Antler lineage periostea. **a** Antlerogenic periosteum; **b** Pedicle periosteum; **c** Antler apical perichondrium; **d** Antler periosteum

It is known now that periosteum covering the frontal crests (presumptive pedicle growth regions) of a prepubertal male deer is the tissue that initiates formation of the pedicle and primary antler [134, 135], and is termed the antlerogenic periosteum (AP; Fig. 2A). Antler regeneration depends on the pedicle periosteum (PP; [136, 137], which envelops the fully grown pedicles (Fig. 2B). Rapid antler elongation is achieved through appositional growth of apical perichondrium (APC; Fig. 2C), where the antler growth centre is located [138, 139]. Shafts of growing antlers are enveloped with antler periosteum (AnP; Fig. 2D), which is distally linked to apical thickened APC and proximally to the PP. The AnP may play a role in antler thickening during the antler growth phase through appositional growth; it also has the potential of tissue repair or partial regeneration of antlers in the event of a mechanical wounding [140]. Overall, deer antler biology is essentially the biology of periosteum/perichondrium.

### The antlerogenic periosteum (AP)

**Fig. 3:**
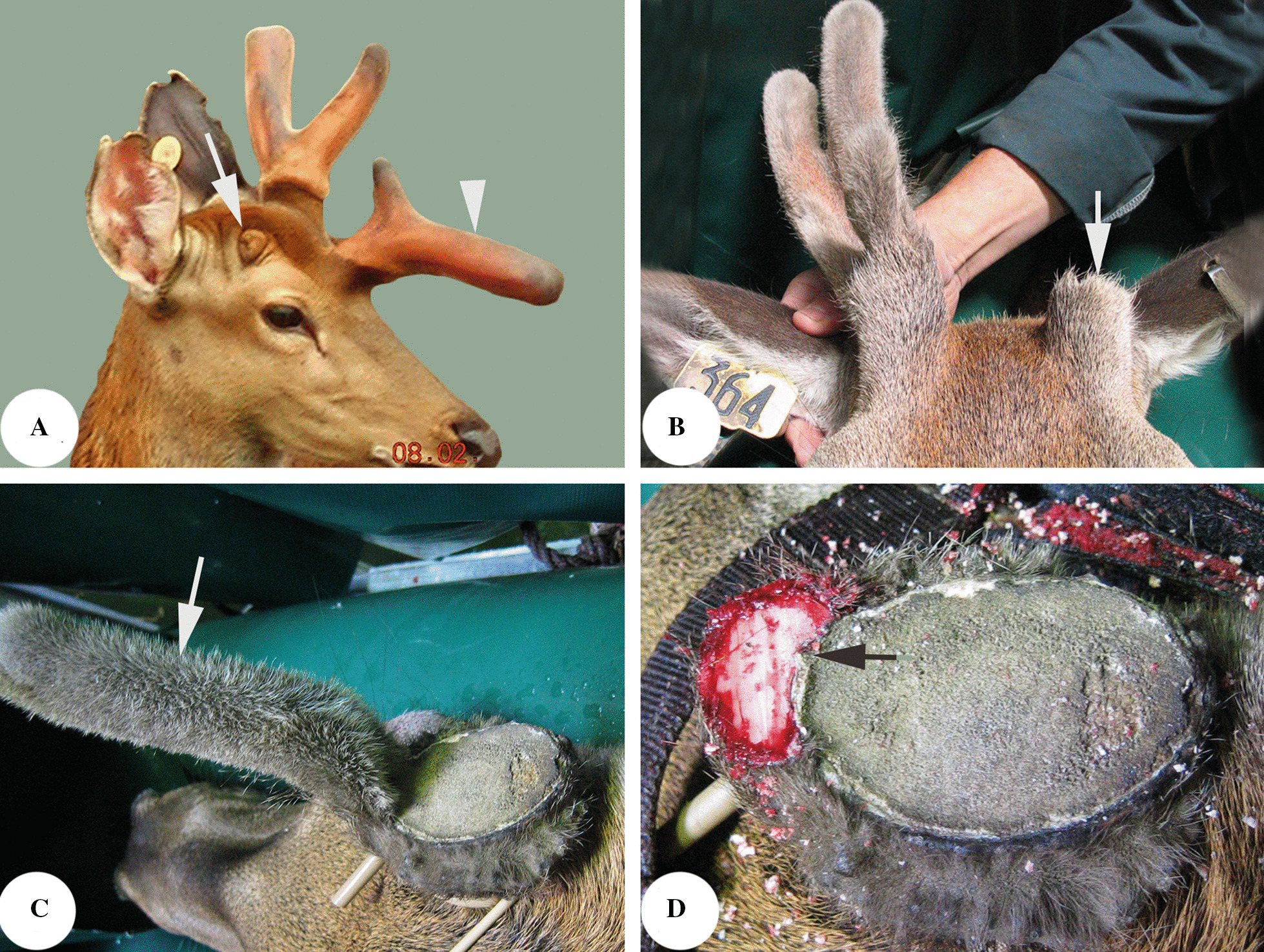
Roles of the different antler-lineage periosteum in antler development. **a** Antlerogenic periosteum (AP), the tissue for pedicle and first antler formation, note that deletion of the AP abrogates future pedicle and antler formation (arrow) and subcutaneous transplantation elsewhere on the deer body induces ectopic antler formation (arrowhead); **b** Pedicle periosteum (PP), the tissue for antler regeneration, note that deletion of the PP renders the PP-less pedicle failure of regenerating antlers (arrow) **c** Antler periosteum (AnP), the tissue for partial antler regeneration/antler tissue repair, note that a spike antler (arrow) is regenerated peripherally around an antler base created after the removal of the antler in its growth phase. **d** The spike antler remanent (arrow) after removing the spike antler, which reveals that the spike antler is regenerated from the AnP of the antler base

The AP is the tissue responsible for the histogenesis of pedicles and primary antlers; this has been demonstrated through surgical manipulation. Deletion of the AP abrogates pedicle and antler formation, whereas autologous transplantation of AP elsewhere on the deer body induces formation of ectopic pedicles and antlers [135, 141]; Fig. 3A). Similarly, transplantation of the AP on immune-deficient animals (such as nude mice) induces formation of xenogeneic antlers [142]. The AP is much thicker than that of the immediate adjacent facial periosteum (FP; [129]), and its cambium layer is three times thicker (e.g. 129 μm vs 35 μm). Ultrastructurally, PDCs from the cambium layer of the AP (aPDCs), prior to pedicle initiation, are spindle-shaped and inactive. A notable feature at this stage is the presence of abundant intracellular glycogen, which renders aPDCs more akin to embryonic osteoblasts. The most striking attribute of the aPDCs at the initial pedicle growth stage is the existence of intracellular mature periodic collagen fibers, which may reflect the unusually high demand for collagen during that period [143]. Interestingly, collagen fibers in the fibrous layer of the hyperplastic AP at this stage exhibit regular waves [129], and cell culture experiments have demonstrated that the proliferative response of fibrous layer PDCs to mitogens (fetal bovine serum or IGF1) is stronger than that of cambium layer PDCs at this stage [144], which is probably why the collagen fibers of the fibrous layer form regular waves. What has happened at this stage may effectively prevent termination of pedicle growth precociously.

A combination of deletion and transplantation experiments has demonstrated that the different sub-regions of the AP (anterior, posterior, medial or lateral) are endowed with different morphogenetic memory, which have been described as morphogenetic fields [145]. In this respect, antler development from the original antler growth region could compensate morphologically for the absence of the posterior or lateral sub-region, but not for the absence of the medial or anterior sub-region. On the other hand, ectopic antlers from either the anterior-AP or medial-AP have the potential to form branches. In contrast, only a single or spike antler is formed ectopically from the other two sub-regions. When a piece of AP is peeled off and then rotated 180° before replacing it, the resultant antlers are found to be oriented backwards on the head [146]. Therefore, we can conclude that the AP determines at least both the anterior–posterior and the proximal–distal axes of the antler, although it is not known thus far whether the AP could influence the dorsal–ventral axis as the antler lacks a landmark to distinguish this axis.

Further studies have shown that aPDCs express mesenchymal stem cell (MSC) markers (CD73, CD90, CD105, Stro-1) and some embryonic stem cell (ESC) markers (TERT, Nestin, S100A4, nucleostemin, C-Myc) [147, 148]. Surprisingly, these cells also express some key ESC markers, such as Oct4, Nanog and SOX2. Some signaling pathways are also activated in the proliferating aPDCs, such as PI3K/Akt, ERK/MAPK, p38 MAPK, [147]. The aPDCs are self-renewing [148] and can be cultured in vitro for up to 80 passages without evidence of senescence. Therefore, the aPDCs are termed antler stem cells. Addition of LY294002, an inhibitor of the PI3K/AKT pathway, in vitro significantly decreased the proliferation rate of the aPDCs and essentially caused a collapse of the cytoskeleton in most of the aPDCs [149].

### The pedicle periosteum (PP)

Once pedicle formation is initiated from the frontal crest, the AP no longer exists but is transformed into the pedicle periosteum (PP), which sheathes the shaft of a grown pedicle. As with the other types of periosteum, the PP also comprises two distinct layers: the outer fibrous layer and the inner cambium layer. The thickness of PP falls midway between that of the AP and the FP. Antler regeneration relies fully on the PP [137]. Total PP deletion prior to antler regeneration prevents the pedicle stump from launching the process (Fig. 3B). However, partial deletion of the PP (distal third of a pedicle stump) results in formation of a regenerating antler bud from the distal end of the remaining PP on the pedicle shaft, a site that is well separated from the distal end of the stump from which the antlers regenerate naturally.

It has been claimed that antler regeneration is a stem cell-based epimorphic process and that the stem cells for antler regeneration reside in the PP [150, 151]. Cells in the PP (pPDCs) of a pedicle stump (created following casting of the previous hard antler) at the distal end are in the potentiated state, and once activated (biochemically or mechanically) these pPDCs will start to proliferate and differentiate to form a regenerating antler growth centre, a process that recapitulates that of formation of initial growth centres for the pedicle and the primary antlers by the aPDCs: firstly intramembranous and then endochondral ossification [129]. Transplantation experiments have shown that the PP has essentially lost the ability to induce ectopic antler formation although it is directly differentiated from AP but has gained the potential to fully regenerate antlers periodically [152].

### The antler periosteum (AnP)

The AnP is histologically a continuation of the distal PP but is thinner than the PP and is enveloped by a special pelage (shiny with a sparse population of hair follicles), known as the velvet skin or velvet. The AnP resembles long bone periosteum and lays down bone peripherally via intramembranous ossification during antler growth. Thus, the antlers are normally thicker than the pedicles from which they are derived. In countries where the velvet antlers are considered as a precious traditional medicine, the antler is removed during the growth phase. To protect the antler growth centre for the next year, the antlers are cut at the level around 3 cm above the junction with the pedicle. Usually, the residual antler bases have the potential to partially regenerate antlers (Fig. 3C), but this potential resides only in the AnP of the antler base (Fig. 3D; [140]). Following experimental removal of a growing antler tip, the AnP on the cut plane (the distal AnP) can also regenerate the lost part although smaller than the contralateral intact antler [153–155].

### The antler apical perichondrium (AAP)

The AAP is located apically at the tip of each antler branch and is the centre of antler growth [156]. Histologically, the AAP comprises a reserve mesenchyme (RM), precartilage and cartilage layers [138]. To facilitate cellular and molecular studies of the antler growth centre, we further refined this classification based on morphologically identifiable markers, BrdU labeling and gene expression profiling on the longitudinal cut surface. This classification includes an extra layer, the transition zone, between the precartilage and cartilage layers. The RM layer is further divided into two sublayers: outer and inner, with the inner containing intensely proliferating mesenchymal cells and the outer containing mitotically quiescent cells; these layers/sublayers each have distinctly different gene expression profiles [157]. Therefore, it is the inner sublayer that drives the very rapid antler elongation. The cells of the outer sublayer exhibit stem cell features (mitotic quiescent) and are responsible for replenishing the transiently amplifying cell pool in the inner sublayer when required [157]. Due to the ease by which these layers can be precisely sampled from fresh antler tissues, the approach has been used widely for the identification of novel factors [147, 149, 158–162].

### Regulation

#### Hormonal

Deer antlers are male secondary sexual characters (except reindeer), as such their growth is strictly under the control of androgen hormones. Pedicle initiation in puberty deer is triggered by the increasing level of testosterone and first antler generation from a fully grown pedicle follows as the testosterone level is decreasing [133, 163]. Both antler calcification and the shedding of the velvet skin are caused by a rapidly rising circulating testosterone level. Hard antler casting occurs when testosterone has fallen to very low levels, while low testosterone is permissive of antler regeneration [132, 133].

Surprisingly, the aPDCs do not respond to testosterone (the major form of androgen) or DHT (dihydrotestosterone, a more powerful version) directly in terms of proliferation when cultured in vitro. This is despite the fact that development of the pedicle and the antler is under the control of androgen hormones and the AP is the tissue that gives rise to the pedicle and antler in vivo and the aPDCs contain androgen specific-binding sites [164]. However, these cells do respond and proliferate in response to IGF-1 in a dose-dependent manner [144]. Given that the actions of androgens are more complex than any other steroids [165], further study is required to elucidate the underlying mechanism.

### Nerves

The somatic periosteum requires a nerve supply, particularly sympathetic nerves, for normal homeostasis and growth [42]. It is known that pedicles are innervated with both sympathetic and sensory nerves, but only sensory nerve fibers go up to the antlers [166, 167]. Interestingly, transection of either sensory [168] or sensory plus sympathetic nerves [169] supplying the presumptive pedicle growth region in a male pubertal deer did not affect subsequent pedicle and antler development, although the resultant antlers were smaller than those from the contralateral sham-operated-regions. An elevated level of NGF expression increases sympathetic innervation while NGF depletion results in sympathetic hypo-innervation [46]. Surprisingly, NGF is highly expressed in the growing antler tip (mainly in the smooth muscle of the arteries and arterioles), but high levels of NGF failed to act as a guidance cue for sympathetic nerves to enter the growing antler [170].

### Tissue interaction

Interactions of the AP and PP with their closely associated skin empower the AP to initiate generation and the PP to initiate regeneration of the antler. These are active induction processes where the AP/PP convert the skin from a scalp type to a velvet type; in turn feedback from the induced skin drives the processes of antler generation or regeneration.

For both pedicle and primary antler formation, proliferation of aPDCs in the AP is activated by elevated circulating androgens at the initial stage, as the pedicle is gradually built up through appositional growth. In this process, the overlying skin is pushed up and becomes mechanically stretched (tension); the consequence of this stretching is the development of a close association between the AP-derived tissue and the overlying skin and the initiation of the first antler is activated [171]. This suggests that antler formation requires the aPDCs to interact with the skin cells, and that the close association facilitates this interaction. Further, membrane insertion experiments [172] have confirmed this hypothesis: when an impermeable membrane was interposed between the AP-derived tissue and the overlying skin, antler formation was inhibited; when a semi-permeable membrane (0.45 µm pore) was used, antler formation occurred, albeit with some delay (1 year).

Antler regeneration requires the PP to interact with the enveloping skin. During tissue sampling, we have found that along the longitudinal axis of a pedicle shaft, the degree of association between the pedicle skin and the PP varies: it is seamlessly fused at the distal one-third, but only loosely-linked at the remaining proximal two-thirds [173]. Membrane insertion experiments have shown that the PP in the fused region had acquired the potential to initiate antler regeneration and form a skinless antler, whereas in the loosely-linked region, the PP remained dormant and membrane insertion stops antler regeneration. Therefore, the former is termed potentiated PP (PoPP) and the latter dormant PP (DoPP) [174]. These results imply that the PP requires interaction with the skin before it can initiate antler regeneration, and that the close association with the pedicle skin facilitates this interaction.

### Possible implications and applications

Overall, antler-lineage periostea are unique in that they: (a) have the ability to respond to androgen hormones to initiate cartilage/bone tissue formation; (b) interact with the closely associated skin to launch development of a postnatal organ (pedicle and primary antler) and full regeneration of mammalian organs (subsequent antlers); and (c) react to potent growth factors to drive bony antler elongation at an unprecedented growth rate.

There may be potential to translate these unique attributes of the antler-lineage periostea for medical use. Specifically, we can make some predictions based on the findings: (1) a unique model for studying the interactions between the grafted tissue and the host environment during initial formation and maintenance of xenogeneic organs. Thus far, we have successfully established a nude mouse model through transplantation of the AP tissue for this purpose. Recently, we carried out single cell sequencing for these nude mouse xenogeneic antlers, and found that besides endothelial and immune cells that all came from the hosts (nude mice), a small amount of cartilaginous and bone cells (1–2%) were also derived from the hosts and left the rest of them (98–99%) from the grafted AP tissue (Wang et al., unpublished), suggesting that the grafted tissue is fully integrated with the host systems for growth and maintenance. Revealing the mechanism underlying this full integration may help to alleviate severe rejection of organ transplantation in clinics. (2) A unique model for studying limb regeneration including amputated human legs and arms. Studies from the model systems (antlers and newt limbs) demonstrated that successful epimorphic regeneration relies on the potent proliferation potential of the distal periosteal cells, and the efficient interactions between the wound epidermis and the mesenchymal tissue on the surface of a leg stump. Based on these assumptions, we have successfully induced partial regeneration of the amputated rat legs through removing the interposing muscle layer and empowering the distal periosteal cells more potent proliferation potential (via delivering relevant genes). In the antler model, although there is a dermal layer interposing the wound epidermis and the underlying mesenchymal tissues, we found that the interactions between wound epidermis and the mesenchymal tissue can be relayed by hair dermal papilla cells [175]. We believe further refinement along these lines, interactions between grafted tissue and host environment would be effectively investigated; and quality and quantity of the appendage regeneration in mammals would be greatly improved, which will eventually benefit humans.

## Data Availability

No applicable.
